# Micro RNA in Exosomes from HIV-Infected Macrophages

**DOI:** 10.3390/ijerph13010032

**Published:** 2015-12-22

**Authors:** William W. Roth, Ming Bo Huang, Kateena Addae Konadu, Michael D. Powell, Vincent C. Bond

**Affiliations:** Department of Microbiology, Biochemistry and Immunology, Morehouse School of Medicine, Atlanta, GA 30310-1495, USA; mhuang@msm.edu (M.B.H.); kateenalj@gmail.com (K.A.K.); mpowell@msm.edu (M.D.P.); vbond@msm.edu (V.C.B.)

**Keywords:** HIV-1, exosomes, microRNA, microarray, qPCR

## Abstract

Exosomes are small membrane-bound vesicles secreted by cells that function to shuttle RNA and proteins between cells. To examine the role of exosomal micro RNA (miRNA) during the early stage of HIV-1 infection we characterized miRNA in exosomes from HIV-infected macrophages, compared with exosomes from non-infected macrophages. Primary human monocytes from uninfected donors were differentiated to macrophages (MDM) which were either mock-infected or infected with the macrophage-tropic HIV-1 BaL strain. Exosomes were recovered from culture media and separated from virus particles by centrifugation on iodixanol density gradients. The low molecular weight RNA fraction was prepared from purified exosomes. After pre-amplification, RNA was hybridized to microarrays containing probes for 1200 miRNA species of known and unknown function. We observed 48 miRNA species in both infected and uninfected MDM exosomes. Additionally, 38 miRNAs were present in infected-cell exosomes but not uninfected-cell exosomes. Of these, 13 miRNAs were upregulated in exosomes from HIV-infected cells, including 4 miRNA species that were increased by more than 10-fold. Though numerous miRNA species have been identified in HIV-infected cells, relatively little is known about miRNA content in exosomes from these cells. In the future, we plan to investigate whether the upregulated miRNA species we identified are increased in exosomes from HIV-1-positive patients.

## 1. Introduction

Micro RNAs (miRNA) are short (20–22 nt) non-coding RNA molecules which are generated from larger precursors, and typically act by binding to 3’ untranslated regions of mRNA transcripts [1]. Thus, they are able to regulate gene expression at the post-transcriptional level. A number of studies have sought to clarify the role of miRNA in HIV disease [2,3,4]. The levels of a number of different host cell miRNA species have been reported to increase or decrease after HIV-1 infection [4,5]. Though individual miRNAs have been characterized in HIV-1 infection, the roles of specific miRNAs have been difficult to assess. Some miRNAs are thought to be involved in mediating immune suppression or in establishment of viral latency [6]. 

Our group has focused on investigating the role of exosomes released during HIV infection [7,8,9,10]. These extracellular vesicles (90–105 nm) are known to carry both protein and RNA, including miRNA. We wanted to investigate whether specific miRNAs are enriched in the exosomes generated by HIV-1 infected cells. For these experiments we used monocyte-derived macrophages (MDM) from uninfected donors. Macrophages have been shown to release large numbers of exosomes after infection with HIV-1 [7]. More importantly, these cells are usually infected by C-C chemokine receptor type 5 (CCR5)-tropic HIV-1 variants, which are the viruses most likely to be present during the early stages of HIV-1 infection. We report the results of a survey of the miRNA content in exosomes released by primary MDM infected with macrophage-tropic HIV-1 BaL. Several miRNAs were significantly increased in exosomes from HIV-infected cells, including a set of four miRNAs that were expressed at very high levels relative to mock-infected cells.

## 2. Methods

### 2.1. Isolation and Culture of Primary Human Cells

Blood samples were obtained with the assistance of Dr. Francois Villinger, Emory University. Donors gave informed consent, and the study was approved by the institutional review boards of Emory University and Morehouse School of Medicine. Blood was collected by venipuncture from HIV-seronegative donors as described previously [9,10] and centrifuged in order to separate plasma and cellular components. Peripheral blood mononuclear cells (PBMCs) were maintained at 37 °C in Roswell Park Memorial Institute Medium 1640 (RPMI-1640) supplemented with 20% heat-inactivated exosome depleted fetal bovine serum, streptomycin (100 U/mL), L-glutamine (2 mM), HEPES-buffered saline solution (10 μM), and IL-2 (20 U/mL). Exosome-depleted serum was prepared by diluting fetal bovine serum 1:4 with RPMI medium and centrifuging 16 h at 100,000× *g*. The 100,000× *g* supernatant was then filtered through a 0.45 μm membrane into a sterile 500 mL bottle. 

### 2.2. Monocyte Differentiation and Infection of Macrophages with HIV-1 BaL

The macrophage-tropic HIV-1 BaL strain was obtained through the National Institutes of Health (NIH) AIDS Reagent Program. Primary donor PBMC were cultured as described above for 48 h. The monocyte cells in the cultures were stimulated to differentiate by addition of 200 nM phorbol myristoyl acetate (PMA) for 4 h, after which the culture medium and non-adherent cells were removed and replaced with fresh medium. The remaining adherent cells (monocyte-derived macrophages) were infected with HIV-1 BAL as described [10] and incubated in the RPMI culture medium for 24 h at 37 °C. After 24 h, the supernatant was removed and fresh medium was added. Every three (3) days, half of the culture medium, containing exosomes and/or virus particles, was removed and replaced with fresh medium. This was continued for 21 days. At the end of the 21-day infection, all the culture supernatants collected over the 21 days of infection were pooled and stored at 4C for processing to isolate exosomes. 

### 2.3. Isolation of Exosomes by Iodixanol Gradient 

Exosomes were separated from HIV virus particles by centrifuging on 6%–18% velocity gradients of iodixanol as described in [9]. Gradient fractions were assayed by for the presence of exosome-specific proteins and for the HIV-1 p24 and Nef proteins (described below). Gradient fractions 2–5, which were positive for exosome markers and negative for HIV proteins were pooled andexosomes were pelleted by ultracentrifugation.

### 2.4. Characterization of Exosomes 

Gradient fractions were assayed for acetylcholinesterase (AChE) activity and HIV-p24 antigen by enzyme-linked immunosorbent assay (ELISA) as described [9,10] Twenty-five microliter aliquots of each fraction were separated by SDS-polyacrylamide (SDS-PAGE) electrophoresis and blotted to membranes as described. Membranes were hybridized with monoclonal antibody to HIV-1 Nef, HIV-1 p24, and human cluster of differentiation (CD) 45 antigens, and polyclonal antibody to human CD 63. Blots were incubated with goat anti- mouse or goat anti-rabbit IgG, labeled with horseradish peroxidase (HRP). Protein bands with HRP activity were visualized by incubating with Luminol substrate.

### 2.5. Preparation of RNA from Exosomes

RNA was extracted from the pelleted exosomes using the miRVana RNA kit (Life Technologies, Carlsbad, CA, USA). The guanidinium-based lysis and phenol/chloroform extraction procedures were done as directed in the protocols supplied with the kit. The final filtration step was included, as suggested by the manufacturer, in order to enrich for low molecular weight (<200 nucleotides in length) RNA species.

### 2.6. Pre-Amplification of RNA and Quantitative PCR (qPCR)

The qPCR experiments were carried out in the laboratories of Life Technologies (Carlsbad, CA, USA). 500 ng RNA for each sample was amplified and labeled using the NCode miRNA amplification system. Labeled RNA was hybridized to NCode V3 Human Microarray cards, which included appropriate human control and reference miRNAs. For each RNA probe, qPCR cycling threshold (CT) values were calculated as suggested by Chen *et al.* [11]. The relative amounts of miRNAs in HIV-infected *vs.* mock infected samples were calculated using the method first described by Litvak [12], where ∆CT is the difference in cycle threshold of the target compared to reference (U6) cycle threshold. The average ∆CT of duplicate samples for mock-infected and HIV-infected were compared. This is the ∆∆CT. The approximate relative concentrations (-fold differences) are expressed by 2^∆∆CT^.

## 3. Results

### 3.1. Exosomes Were Purified from HIV-Infected Monocyte-Derived Macrophages (MDM)

**Figure 1:**
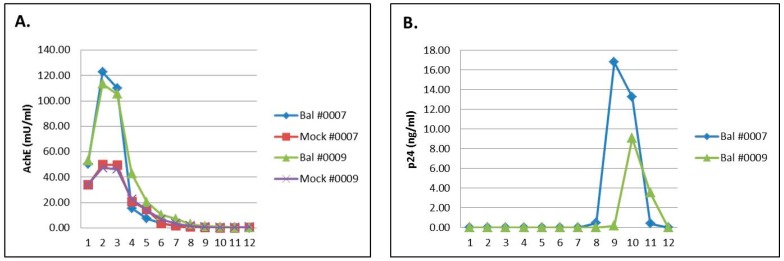
(**A**) Acetylcholinesterase (AchE) activity in iodixanol gradient fractions from HIV-1-infected and mock-infected culture media. HIV-1 virus particles and exosomes were concentrated from culture media by centrifugation and layered on iodixanol gradients. Gradient fractions were individually assayed and AchE activity in milliunits/mL was plotted for each fraction. (**B**) HIV-1 p24 capsid protein concentration in gradient fractions from HIV-1-infected and mock-infected culture media. HIV-1 virus particles and exosomes were concentrated from culture media by centrifugation and layered on iodixanol gradients. Gradient fractions were individually assayed and HIV-1 p24 concentration in ng/mL was plotted for each fraction.

**Figure 2:**
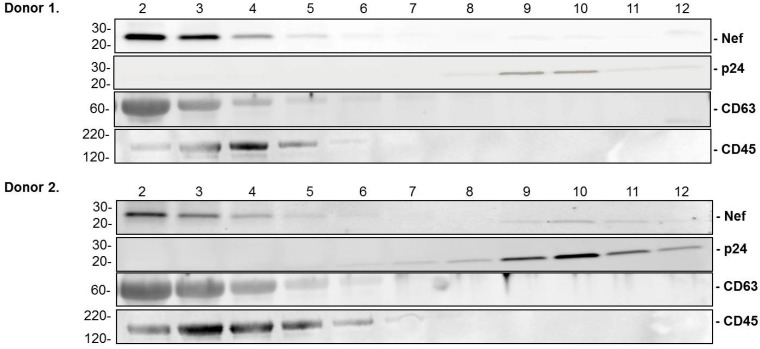
Western blotting of proteins in exosome preparations from HIV-1-infested MDM. The proteins in individual iodixanol gradient fractions 2-12 from HIV-1-infected MDM from donors 1 and 2 were separated by SDS-PAGE and transferred to polyvinylidene fluoride (PVDF) membranes. The membranes were incubated with antibodies to exosomal proteins CD 45 and CD 63 and HIV-1 proteins p24 and Nef.

MDM from two uninfected donors were either mock-infected or infected with HIV-BaL as described above. Duplicate samples for each donor and condition were prepared. The fractions of the iodixanol gradient for each sample were assayed individually for acetylcholinesterase (AchE) activity (Figure 1A) and by Western blotting for cellular antigens CD45 and CD63 (Figure 2), all previously shown to be present in exosomes, but not in HIV-1 particles. 

Gradient fractions were also assayed by ELISA assay for HIV-1 capsid protein p24 (Figure 1B), and by Western blotting for HIV-1p24 and Nef proteins (Figure 2). Analysis of AchE activity and ELISA analysis of HIV-1 p24 in gradient fractions 1–12 is shown in Figure 1. Western blotting for exosomal and viral proteins in gradient fractions 2–12 is shown in Figure 2. Gradient fraction 1 was omitted from the Western analysis. This fraction, corresponding to the sample volume loaded on each gradient, contains AChE as well as other proteins that react with the AChE antibody and which interfere with antibody binding in adjacent fractions. Due to possible contamination by cellular proteins, gradient fraction 1 was not included in pooled exosome samples used for miRNA preparation. 

These assays conclusively show that MDM-derived exosomes were present in fractions 2–5 at the top of the gradients, while virus particles sedimented in gradient fractions 8–12, near the bottom. Thus, the iodixanol gradient procedure yielded exosomes that were free of contamination by HIV particles and cellular components, and which could be used for extraction of exosomal RNA.

### 3.2. Changes in Exosomal miRNA Content Were Observed after HIV-1 Infection

The exosomal RNA preparations from HIV-infected and mock-infected MDM were shipped to the laboratories of Life Technologies, where they were amplified, labeled and hybridized to a microarray panel made up of miRNA-specific probes. The NCode V3 arrays used for these experiments contained probes for more than 1200 miRNA species, including miRNAs from the miRBase database and additional miRNAs identified by deep genomic sequencing [13]. 

The qPCR hybridization results are reported as cycling threshold (CT) values, which have been normalized to CT values for human U6 RNA. The CT value is a measure of the relative concentration of a given RNA. A lower CT value indicates a relatively higher miRNA level in a given sample. The values for the two mock-infected and the two HIV-infected samples were averaged; mock-infected and HIV-infected averages were then compared to determine relative increases or decreases for individual miRNA species. 

**Figure 3:**
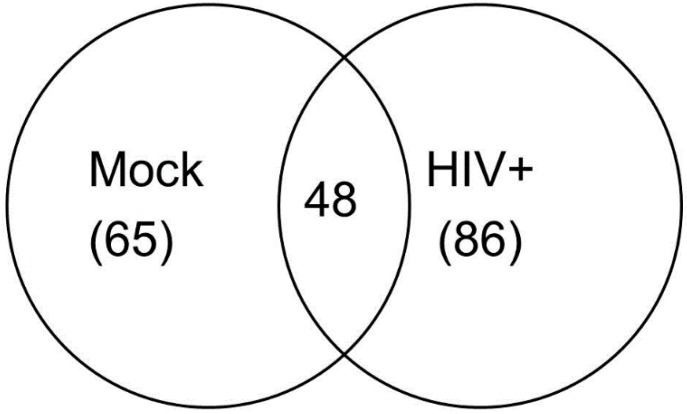
Venn diagram showing the numbers of miRNAs detectable only in mock-infected cell exosomes (65), only in HIV-infected cell exosomes (86), and in at least one exosome sample from both mock- and HIV-infected cells (48).

A total of 86 different miRNA species were detected in at least one of the HIV-1 infected exosome samples. Of these, 48 miRNAs were detected in at least one exosome sample from both HIV-1-infected and mock-infected donor cells (Figure 3). A smaller group of 38 miRNAs were detected in exosomes isolated from HIV-1-infected cells of both donors but not in exosomes from mock-infected cells (Table 1).

**Table 1:** Human miRNAs expressed only in HIV-infected cell exosomes.

miR-106a#	miR-520d-5p
miR-1289	miR-523
miR-136	miR-548a-5p
miR-184	miR-548c-3p
miR-194	miR-601
miR-20a	miR-645
miR-211	miR-758
miR-21	miR-876-3p
miR-30a	miR-891b
miR-34a	miR-106b
miR-361	miR-1247
miR-367	miR-133a
miR-381	miR-16-2#
miR-422	miR-16
miR-425	miR-19b
miR-483	miR-331
miR-487a	miR-372
miR-506	miR-520b
miR-518e	miR-708

Of the 48 miRNAs from both mock-infected and HIV-infected MDM exosomes, only 33 miRNA species were detected in duplicate samples from both donors. Most of these miRNAs were expressed at similar levels in mock-infected and HIV-infected cell exosomes. However, 13 miRNAs were increased by more than 1.5-fold as a result of HIV-1 infection (Table 2). The largest increase was seen in four of the miRNAs (miR-29a, miR-150, miR-518f, miR-875), which were up-regulated by a range of 16-fold to 44-fold in the HIV-infected cell exosomes. These changes were not statistically significant due to the small number of samples analyzed. Interestingly, no exosomal miRNA species were shown to be decreased as a result of HIV-1 infection of MDM.

**Table 2:** CT * values for miRNAs upregulated in HIV-1 infected cell exosomes.

miRNA	1-Infect	2-Infect	Average	1-Mock	2-Mock	Average	∆∆CT ^#^	2^∆∆ CT @^
1243	32.39 *	31.44	31.91	33.23	34.40	33.81	1.9	3.70
1274a	28.75	28.18	28.46	30.18	29.64	29.91	1.45	2.73
150	27.10	28.68	27.89	32.49	33.64	33.07	5.18	36.25
29a	26.48	30.62	28.55	34.19	33.85	34.02	5.47	44.32
302c	18.74	19.91	19.33	21.62	21.95	21.79	2.46	5.50
30e	29.47	31.07	30.27	31.58	33.16	32.37	2.10	4.28
338	30.75	32.82	31.79	34.05	32.91	33.48	1.69	3.22
454	24.88	26.57	25.73	25.67	30.61	28.14	2.41	5.31
518f	20.36	22.24	21.3	25.98	25.94	25.96	4.66	25.28
548a	33.17	32.80	32.99	34.22	33.19	33.71	0.72	1.65
636	20.20	24.00	23.1	26.34	25.69	26.02	2.92	7.50
872	25.72	26.16	25.94	27.09	28.09	37.59	1.65	3.14
875	25.05	25.77	25.41	29.11	29.68	29.39	3.98	15.78

^1 and 2 -infect or -mock refers HIV-infected or Mock-infected samples from donors 1 and 2, respectively. * qPCR cycling threshold—lower CT value indicates higher miRNA expression (see Methods). ^#^ Difference between Avg. HIV-infected and Avg. mock-infected. ^@^ Fold difference (value of 3.70 indicates a 3.7-fold increase in miRNA in HIV-infected samples).

## 4. Discussion

Small (90–105 nm diameter) microvesicles, or exosomes, are secreted from immune cells and have been shown to function in cell-to cell communication [14,15,16]. Our group and others have demonstrated a role for exosomes in HIV-1 infection. In the early stages of HIV infection the predominating virus strains are those that utilize the CCR5 co-receptor for entry into target cells. These virus strains are able to infect monocyte-derived macrophages (MDM), which express CCR5 [17]. We have shown that infection of MDM leads to the release of increased numbers of exosomes into the extracellular space [7], where they play a role in immune activation and may help to establish and prolong the viral infection [10].

MiRNAs are expressed in most cell types and are known to regulate gene expression [1]. However, though there is increasing information regarding possible roles for cellular miRNAs during HIV-1 infection, very little is known about miRNAs packaged in exosomes released by HIV-infected cells. In order to examine the role of miRNA in exosomes during the early stages of HIV infection, we used the macrophage-tropic HIV-1 BaL to infect primary MDM from healthy uninfected human donors. Low molecular weight RNA was prepared from purified exosomes and was hybridized to a comprehensive set of miRNA probes. 

We were able to detect 86 different miRNA species in the exosomes from HIV-1-infected macrophages, though some were detectable in only one of two samples tested. There were 38 miRNA species (Table 1) present in both the HIV-infected samples but not in the mock-infected samples. A small number of miRNAs were seen in mock-infected but not in HIV-infected exosomes. The CT values for these miRNAs are consistent with low levels of expression (not shown). It is possible that they were also present in the infected samples, but in undetectable amounts. As stated earlier, we did not observe any miRNA species that were present at high levels in mock-infected exosomes and were decreased in exosomes from HIV-infected MDM. 

The precise role of miRNA in HIV infection is still unclear. Two of the four miRNAs strongly upregulated in our study, miR-29a and miR-150, were previously reported as being expressed in the cytosol of HIV-1 infected cells [4]. The level of miR-29a was shown to be decreased dramatically in the virus infected cells [2]. Although miR-29a was not previously shown to be present in exosomes, another member of the same miRNA family, miR-29b was seen in exosomes in neural tissues of SIV-infected macaques [18]. In an earlier study, miR-150 was shown to be down-regulated during HIV infection and has been suggested as a possible biomarker related to HIV disease progression [19]. A group of miRNAs, including miR-150, were observed to decrease during differentiation of monocytes to macrophages [20].

Other clues to miRNA roles in HIV infection may come from previously assigned cellular functions. Recently, both miR-29a and miR-150 were proposed to act as tumor suppressors [21,22]. Also, miR-518f was shown to be increased in HIV-infected lymphocytes [23], where a three-fold increase was seen as a result of virus infection, as compared to a 25-fold increase seen by us in HIV-infected MDM exosomes. In addition, miR-518f was shown to be expressed in normal T-lymphocyte exosomes, where it is thought to be involved in the immune response [24]. The miR-518f species is also postulated as being involved in DNA replication, recombination and repair pathways. MiR-875 has not been previously reported to be associated with HIV, and its function remains unknown.

## 5. Conclusions

We were able to detect more than 80 different miRNAs in exosomes from primary donor macrophages infected by the macrophage-tropic HIV-1 BaL strain. Four miRNA species were very strongly upregulated, increasing by amounts ranging from 15- to 40-fold. Two of these, hsa-miR-29a and hsa-miR-150, are known to be down regulated in HIV-1 infected cells, although their roles in exosomes remain to be determined.

We propose that as a result of HIV-1 infection, a group of cellular miRNAs are preferentially directed to exosomes, thereby decreasing intracellular amounts. Directed insertion of miRNA into exosomes was also proposed by Aqil *et al.* [25] who compared cellular and exosomal miRNAs in a monocyte cell line stably transfected with the HIV-1 nef gene. Interestingly, our group and others have shown that HIV-1 Nef stimulates exosome formation and directs its own insertion into exosomes [8,26,27]. Both miR-29a and miR-150 were among a group of miRNAs directed into exosomes [28], and miR-29b was also shown to be directed into exosomes [28], though we did not detect it in our exosome samples. The present study shows changes in exosomal miRNA resulting from infection of differentiated primary monocytes by HIV-1. This builds on the study of the HIV-1 infection of a monocyte cell line [25] and supports the hypothesis that exosomes are a means of shuttling RNA between cells and decreasing miRNA species in virus-infected cells.

Earlier studies coupled with the data presented here suggest that during HIV infection, miRNA species are packaged into exosomes, either as a way of decreasing the intracellular content of specific miRNAs or to release miRNAs for uptake by neighboring non-infected cells. More studies are required, especially to determine whether exosomes containing specific miRNAs are present in the plasma of HIV-infected individuals.
